# Penile Implants among Prisoners—A Cause for Concern?

**DOI:** 10.1371/journal.pone.0053065

**Published:** 2013-01-11

**Authors:** Lorraine Yap, Tony Butler, Juliet Richters, Eva Malacova, Handan Wand, Anthony M. A. Smith, Luke Grant, Alun Richards, Basil Donovan

**Affiliations:** 1 Kirby Institute, University of New South Wales, Sydney, New South Wales, Australia; 2 School of Public Health and Community Medicine, University of New South Wales, Sydney, New South Wales, Australia; 3 School of Population Health, University of Western Australia, Perth, Western Australia, Australia; 4 Australian Research Centre for Sex, Health and Society, La Trobe University, Melbourne, Victoria, Australia; 5 Offender Services and Programs, New South Wales Department of Corrective Services, Sydney, New South Wales, Australia; 6 Communicable Diseases Unit, Chief Health Officer Branch, Queensland Health, Brisbane, Queensland, Australia; 7 Sydney Sexual Health Centre, Sydney Hospital, Sydney, New South Wales, Australia; Rollins School of Public Health, Emory University, United States of America

## Abstract

**Background:**

We report the prevalence of penile implants among prisoners and determine the independent predictors for having penile implants. Questions on penile implants were included in the Sexual Health and Attitudes of Australian Prisoners (SHAAP) survey following concerns raised by prison health staff that increasing numbers of prisoners reported having penile implants while in prison.

**Methods:**

Computer-Assisted Telephone Interviewing (CATI) of a random sample of prisoners was carried out in 41 prisons in New South Wales and Queensland (Australia). Men were asked, “Have you ever inserted or implanted an object under the skin of your penis?” If they responded Yes: “Have you ever done so while you were in prison?” Univariate logistic regression and logistic regression were used to determine the factors associated with penile implants.

**Results:**

A total of 2,018 male prisoners were surveyed, aged between 18 and 65 years, and 118 (5.8%) reported that they had inserted or implanted an object under the skin of their penis. Of these men, 87 (73%) had this done while they were in prison. In the multivariate analysis, a younger age, birth in an Asian country, and prior incarceration were all significantly associated with penile implants (p<0.001). Men with penile implants were also more likely to report being paid for sex (p<0.001), to have had body piercings (p<0.001) or tattoos in prison (p<0.001), and to have taken non-prescription drugs while in prison (p<0.05).

**Conclusions:**

Penile implants appear to be fairly common among prisoners and are associated with risky sexual and drug use practices. As most of these penile implants are inserted in prison, these men are at risk of blood borne viruses and wound infection. Harm reduction and infection control strategies need to be developed to address this potential risk.

## Introduction

Penile implants are inert objects placed beneath the skin of the penis through an incision and are variously referred to as Yakuza beads, pearls, penile implants, penile beads, penile nodules, penile inserts, speed bumps, and penile marbles in the English literature [1]–[6]. Objects placed underneath the skin of the penile shaft may include ball bearings, plastic beads made from toothbrushes, glass, metal pellets, silicon, precious metals, marbles or pearls [1] [7]–[8]. The practice is distinct from inflatable prostheses or semi-rigid bars to treat impotence.

Reports of penile implanting usually appear in the form of case studies in the clinical literature or in qualitative research studies [4] [9]–[15]. To the best of our knowledge, penile implants have rarely or never been researched as part of any population based epidemiological study of prisoners but, as one group of US clinicians concluded after seeing several cases of penile implant mishaps occurring in prison, *“penile modification by self-placement of foreign bodies is not a rare practice among some members of the incarcerated population*” [15].

Penile implants have been traditionally reported among men of Asian (77%, 27 out of 35 case reports and studies) and Slavic (18%) origin with few from Western cultures (3%) [2]. The practice is also said to be more common among seamen, prisoners, drug addicts, the military, and those from lower socio-economic backgrounds [3] [9]–[10] [16]–[17]. Anthropologists have documented that inserting objects under the penile skin, as part of cultural traditions, has been practised for centuries in the Asia and Pacific region. Men in North and Southeast Asia have a long history of inserting bells, balls and other irregular objects under the skin of their penis. In Australia, some Aboriginal men have been reported to observe a tradition of placing small stones inside penile incisions [1].

Some men believe that penile implants enhance the sexual pleasure and make them unforgettable to women, and in some cases, discourage men from practicing sodomy [1] [6]–[8]. Penile implants were also reportedly used as “charms” and body magic in Asian cultures against the culture-specific syndrome, *koro*, or the fear that the penis will shrink and retract inside the body causing death [1]. Studies among different groups suggests that penile implants were used to increase sexual confidence, as self-ornamentation, to reinforce masculinity and as a marker for attaining manhood, as a symbol of affiliation to a certain group, practiced in partner sadism, for revenge by deliberately causing harm through sex or rape of sex workers and women with multiple partners or among women who had refused sexual invitations, or were adopted as a result of peer pressure or curiosity [1]–[2] [4] [6] [8]–[11] [13] [18]. In Bali, men were encouraged by their peers to have penile implants to increase their chances of obtaining free oral or anal sexual services from commercial sex workers and for a better sexual experience. It is believed that penile implants produce more friction during sexual intercourse with sex workers [11].

The origins of the custom among prisoners of inserting penile implants date back to the 18th century among the *yakuza* (Japanese gangsters) as a demonstration of their loyalty to the clan [19]–[21]. A survey in a Japanese detention centre found a prevalence of 22% (28/130) of men with penile implants, and prison records showed that most of the men belonged to the *yakuza* [20]–[21]. Case reports and studies of prisoners and ex-prisoners in Eastern Europe, the United States, Papua New Guinea and Indonesia suggest that this population may be gradually adopting the practice [4] [6] [10] [13] [15].

In prison, beads made from spoons, toothbrushes, dominoes or chopsticks have been reported as being inserted [4] [7] [15] [20]. Other penile implants included beads made from melted toothpaste tube caps, buttons, rubber erasers, dice, or deodorant roller balls [4] [7] [20]. Wardi's (2011) study of Indonesian prisoners suggests that the practice has become inter-generational inside prison. Making, polishing and subcutaneously inserting the penile beads in the foreskin is one method for prisoners to stimulate themselves and prevent boredom in a place with few recreational activities and, at the same time, provide them with an income from selling and inserting the finished penile beads into other prisoners [13]. Prison officers in Papua New Guinea also believe that the practice is the result of boredom and a way for prisoners to pass the time in prison [6].

In US prisons, hygiene was reported to be problematic because requests for antiseptics could lead to intense questioning from prison health staff. Focus groups with prisoners found that they were reluctant to present themselves for treatment at prison clinics if their genitals became infected for fear of being punished by prison authorities. Moreover, they would be pressured by authorities to identify the individuals who performed the operation which would bring direct harm to themselves inside jail for “snitching” [4]. In Indonesia, despite threats of punishment from prison authorities if caught, continuing to implant penile nodules and beads was seen as a form of political resistance against the domination of the prison establishment [13].

Penile inserts are known to result in a number of medical complications [3]–[4] [10] [15]–[17] [20] [22]–[24]. Reports include penile oedema and erythema, throbbing pain and inflammation after insertion, and penile infections and abscesses [5] [8] [15]. Artificial modification of the penis can result in sexual difficulties such as erectile dysfunction or partner vaginal trauma, and can sometimes prevent penetration [5] [8] [25]. Women, including wives and female sex workers, have complained of pain during intercourse [5] [11] [25], and implants have been known to cause bleeding and damage to their vagina and cervix [6].

Penile implants can possibly increase the transmission of HIV, other sexually transmissible infections (STIs) and blood borne viruses as they can contribute to condom breakage, leakage, and incorrect fit [12]. Transmission of blood borne viruses can occur if the person who is performing the incision is exposed to the other person's blood [4] [11]. Interviews with Indonesian fishermen further suggest that STI and blood borne viruses transmission could occur when men who have recently had penile implants engage in intercourse with sex workers before their incision wounds have healed [11].

In the Sexual Health and Attitudes of Australian Prisoners Study (SHAAP) study, we added questions on penile implants to determine how widespread this practice was among prisoners in New South Wales (NSW) and Queensland (Qld) as prison health staff had expressed concerns about penile implants during the development of the SHAAP study.

We report, for the first time, the prevalence of penile implants in a large sample of Australian prisoners and describe factors associated with penile implants.

## Methods

The Sexual Health and Attitudes of Australian Prisoners (SHAAP) survey interviewed randomly sampled men and women prisoners in New South Wales (September 2006 to December 2006) and Queensland (September 2007 to June 2008) [26]–[28]. The survey utilised a computer-assisted telephone interview (CATI) format [29]. To the best of our knowledge, this is the first time that this [CATI] approach has been used to screen a prisoner sample in an epidemiological survey.

A total of 1,118 men in NSW and 900 in Queensland, aged between 18 and 65 years, were surveyed and asked, “Have you ever inserted or implanted an object under the skin of your penis?” (If necessary, interviewers clarified: Things like ball bearings, pieces of plastic, metal or other objects, not including piercing.) (Yes/No). If they responded Yes: “Have you ever done so while you were in prison?” (Yes/No).

The overall response rate to the survey was 82.9% (82.6% for men and 82.9% for women) in NSW and 75.3% (76.0% for men and 71.2% for women) in Queensland. Prisoners in these two states account for approximately 60% of all prisoners in Australia [27].

### Recruitment

Potential participants were randomly selected from a list of all inmates at a particular prison provided by the two Departments of Corrective Services several days prior to interviews [27]. Lists were imported into SPSS 15 [30] and a random sample drawn so as to achieve the target sample size at each prison (40% of the female inmate population and 13% of the male population). The quota of prisoners sampled at each facility was proportional to the size of the prisoner population at that site. The sample was generated at the individual prison as close to the interview period as possible to minimise loss due to releases, transfers to other prisons, and other types of absences from the prison (e.g. home visits, court appearances, and escapes). All prisons in each state were included in the survey apart from a small number (<1% of the total prisoner population in these jurisdictions) of remote work camps. This was due to the high cost of travelling to these sites and other logistical difficulties such as a lack of telephone access.

Those selected were provided with a verbal explanation of the study by a recruiter attached to the research team, not the custodial authorities, and provided with a printed information sheet and consent form. The information sheet explained that certain demographic and criminographic information (e.g. offence type and sentence length) would be obtained from the Departments of Corrective Services, participation was voluntary, and only those who provided written consent could participate. Participants were reassured that the phone call would not be electronically eavesdropped by the custodial authorities and that they could withdraw at any time without consequence.

Those inmates who were unavailable, ineligible, or refused to participate were replaced by randomly selected replacements. A unique study code was generated for each inmate to avoid any possibility of identification. Once the survey information had been linked to the demographic and criminographic information provided by the Departments of Corrective Services, the identifying information was removed.

Following the completion of the interview, participants were de-briefed by the recruiter with the option of organising a referral to a counsellor or the health clinic in the prison if needed. Each participant received AUD$10 as compensation for time lost while engaged in paid work in the prison; this was paid into the participant's prison account.

### Interviews

Telephone interviews were conducted by a private social market research company located in central Sydney. Most interviewers were women but the option existed to be interviewed by a male interviewer (nobody requested this option). Training was provided to the interviewers by the senior researchers but most interviewers were highly experienced in undertaking social and health research on sensitive topics. The prison interviews took place in a private space (often a legal visits room or consulting room in the health clinic) and lasted on average about 30 minutes (range: 19 to 60 minutes). The setting for the interview was chosen to provide privacy for the participant with no custodial officer or researcher present in the room during the interview. The telephone had a headset and microphone attached and, at the insistence of the custodial authorities, was designed such that outside calls could not be made on the device.

### Questionnaire

This study utilised a modified version of the questionnaire used in the Australian Study of Health and Relationships (ASHR) Survey with minor modifications of wording to allow for the lower literacy of this sample and the addition of further sections on experiences in prison [27] [31].

The survey questionnaire covered a broad range of sexual health issues including: sexual identification, sexual attraction, first sex experience (e.g. age, relationship, condom use), same and opposite sex relationships (e.g. number of partners and condom use), relationship prior to prison, impact of imprisonment on relationship, consensual and non-consensual sex while in prison, expectations of sexual exclusivity while prison, contraception, last sexual experience before prison, pregnancy, masturbation and esoteric practices, sexual difficulties, sex work and paying for sex, risk behaviours prior to and after prison, physical assault in prison, overall physical (SF-12) [32] and mental health (K6) [33], drug and alcohol use, attitudes to sex, and knowledge of STI transmission [27] [31].

### Statistical analysis

Univariate logistic regression and forward stepwise multivariate logistic regression were used to determine the independent predictors for penile implants. Marginally significant variables with p<0.10 in univariate analysis were included in the multivariate analysis. Variables with p<0.05 were retained in the final multivariate logistic model. All analyses were performed using Stata 10.0 (College Station, TX).

### Ethics

Ethical approval was granted by the NSW Justice Health Human Research Ethics Committee (GEN5/05), the University of NSW Human Research Ethics Committee (HREC 05045), the NSW Department of Corrective Services Ethics Committee (Ref 05/0882) and the Qld Corrective Services Research Committee. All participation was voluntary and respondents who declined to participate or otherwise did not participate were eligible for treatment and were not disadvantaged in any way.

## Results

Of the 2,018 male inmates surveyed, 118 (5.8%) reported that they had inserted or implanted an object under the skin of their penis. Of these, 87 (73%) had this done while they were in prison.

### Univariate analysis

#### Sociodemographic characteristics

Male prisoners who had penile implants were more likely to be younger (aged <35 years), to have been born in Asia (in decreasing order of frequency; Vietnam, Philippines, Malaysia, and Hong Kong), to have a history of prior incarceration, and to have been in prison for 2 or more years over their lifetime (Table 1).

**Table 1:** Comparison of male prisoners in New South Wales and Queensland with and without penile implants.

Characteristic	Prisoners with penile implants		Prisoners without penile implants		Univariate			Multivariate		
	*No.*	*%*	*No.*	*%*	OR (95% CI)		p Value	OR (95% CI)		p Value
**Gender**										
Male	118	*5.85*	1900	*94.15*						
**Age Group**										
<25	33	*27.97*	433	*22.79*	3.04	(1.71–5.41)	**<0.001**	4.58	(2.46–8.51)	**<0.001**
25–34	66	*55.93*	709	*37.32*	3.71	(2.21–6.24)	**<0.001**	2.88	(1.66–4.98)	**<0.001**
35+	19	*16.10*	758	*39.89*	ref					
**Aboriginal Descent**										
Yes	27	*22.88*	414	*21.79*	1.00	(0.64–1.55)	0.984			
No	91	*77.12*	1389	*73.11*	ref					
Unknown			97	*5.11*						
**Education**										
No secondary school	16	*9.17*	169	*8.89*	1.52	(0.87–2.63)	0.139			
Secondary school only	98	*82.66*	1570	*82.63*	ref					
Post-secondary school	3	*7.73*	153	*8.05*						
**Marital Status**										
Married	5	*4.24*	174	*9.16*	ref					
Never married	99	*83.90*	1398	*73.58*	2.46	(0.99–6.14)	0.053			
Widowed, separated or divorced	14	*11.86*	327	*17.21*	1.49	(0.53–4.21)	0.451			
Refused			1	*0.05*						
**Sexual Identity** #										
Heterosexual	111	*94.07*	1808	*95.16*	ref					
Bisexual	6	*5.08*	56	*2.95*	1.75	(0.74–4.14)	0.206			
**Region of Birth**										
Australia	94	79.66	1657	*87.21*	ref					
Asia	12	*10.17*	80	*4.21*	2.51	(1.32–4.77)	**0.005**	5.52	(2.68–11.37)	**<0.001**
Other	12	*10.17*	163	*8.58*	0.82	(0.44–1.52)	0.529			
**Not first time in prison**	102	*86.44*	1122	*59.05*	4.42	(2.59–7.55)	**<0.001**	3.00	(1.68–5.36)	**<0.001**
**Total time in prison**										
<2 years	15	*12.71*	779	*41.00*	ref					
2 years or more	103	*87.29*	1115	*58.68*	4.80	(2.77–8.31)	**<0.001**			
**Time served of current sentence**										
<2 years	78	*66.10*	1330	*70.00*	ref					
2 years or more	40	*33.90*	568	*29.89*	1.20	(0.81–1.78)	0.362			
**Told by a health professional that you have or have had an emotional or mental health problem** *	44	*37.29*	640	*33.68*	1.17	(0.80–1.72)	0.425			
**Ever had same sex experience**	19	*16.10*	244	*12.84*	0.77	(0.46–1.28)	0.309			
**Ever had sexual contact with another prisoner**	19	*16.10*	125	*6.58*	2.71	(1.61–4.58)	**<0.001**			
**Consented to sexual contact in prison**										
Yes	17	*14.41*	107	*5.63*	1.19	(0.25–5.68)	0.826			
No	2	*1.69*	15	*0.79*	ref					
Sort of			3	*0.16*						
**Ever been paid for sex**	54	*45.76*	664	*34.95*	1.56	(1.07–2.27)	**0.020**	2.62	(1.58–4.36)	**<0.001**
**Ever paid for sex**	28	*23.73*	143	*7.53*	3.81	(2.41–6.01)	**<0.001**			
**Sexual frustration in prison**	86	*72.88*	1039	*54.68*	2.21	(1.46–3.36)	**<0.001**			
**Ever masturbated in prison**	103	*87.29*	1489	*78.37*	3.94	(1.44–10.81)	**0.008**			
**Erection problems**	32	*27.12*	472	*24.84*	1.12	(0.74–1.71)	0.592			
**Any STI** ∧	30	*25.42*	422	*22.21*	1.19	(0.78–1.83)	0.417			
**Hepatitis**										
Hepatitis A	2	*1.69*	50	*2.63*	0.64	(0.15–2.64)	0.533			
Hepatitis B	3	*2.54*	86	*4.53*	0.55	(0.17–1.77)	0.319			
Hepatitis C	54	*45.76*	447	*23.53*	2.78	(1.90–4.06)	**<0.001**			
**Any pain problem with penis**	14	*11.86*	169	*8.89*	1.39	(0.78–2.48)	0.268			
**Ever had body piercing**	72	*61.02*	785	*41.32*	2.22	(1.52–3.25)	**<0.001**			
**Ever had body piercing while in prison**	32	*27.12*	79	*4.16*	7.15	(4.25–12.02)	**<0.001**	4.31	(2.53–7.32)	**<0.001**
**Ever tattooed**	100	*84.75*	1113	*58.58*	3.93	(2.36–6.54)	**<0.001**			
**Ever tattooed while in prison**	70	*59.32*	435	*22.89*	3.63	(2.33–5.66)	**<0.001**	2.79	(1.79–4.36)	**<0.001**
**Ever taken illicit drugs**	109	*92.37*	1490	*78.42*	3.32	(1.67–6.60)	**<0.001**			
**Ever taken illicit drugs in prison**	61	*51.69*	489	*25.74*	3.08	(2.11–4.48)	**<0.001**	1.56	(1.03–2.38)	**0.037**
**Ever injected drugs in prison**	30	*25.42*	210	*11.05*	2.74	(1.76–4.24)	**<0.001**			

# None of the prisoners with penile inserts identified as gay or homosexual although one prisoner reported ‘other’ sexual identity.

* Additional wording in the survey: [Interviewer guide indicated that such conditions as depression, schizophrenia, manic depressive, anxiety, personality disorder, alcohol dependence, drug dependence and ADD/ADHD should be included].

∧ Yes to any of the following: chlamydia, genital herpes, syphilis, gonorrhoea, non-specific urethritis (NSU), genital warts (including anal warts), trichomoniasis.

#### Physical, mental and emotional health and wellbeing

No significant differences were detected between those with and without penile implants in relation to self-reported erectile problems or pain in the penis. Nor were there differences with regard to self-reported mental health problems (i.e. current history of depression, schizophrenia, manic depression, anxiety, personality disorders, and ADD/ADHD), or alcohol and drug use (Table 1).

#### Sexual identity and behaviours

No difference was found in terms of the proportion of those with and without implants in terms of sexual identity (homosexual/gay, bisexual or heterosexual/straight). None of those with penile implants identified as homosexual. Men with penile implants, however, were more likely to have had sexual contact with another inmate in the past and to report having been diagnosed with hepatitis C. Those with penile implants were more likely to report sexual frustration inside prison than those without implants (Table 1).

### Multivariate analysis

In adjusted analysis, a younger age, birth in an Asian country, and prior incarceration remained significantly associated with penile implants. Also, men with penile implants were more likely to have been paid for sex, to have had body piercings or have tattoos while in prison, and to have taken non-prescription drugs while in prison (Table 1).

## Discussion

This paper is the first population-based epidemiological study to show the extent of penile implants among prisoners, and that most (73% of those with penile implants) report having had them inserted whilst in prison. This confirms case reports among clinicians who suggest that penile implants are not uncommon among incarcerated populations [9]–[10] [14]–[15]. In this study, the likelihood of having a penile implant increased with younger age, possibly suggesting that the incidence of the practice is increasing among prisoners. Having an Australian Indigenous background was not significantly associated with penile implants. Men with penile implants had a higher probability of using illicit drugs in prison, of being paid for sex, and having piercings and tattoos in prison, indicating riskier lifestyles than the average prisoner.

Apart from this study, there is a lack of knowledge on penile implants in Australian prisons. It is not known how widespread the practice is across all jurisdictions, how the practice is performed in prison, its impact on disease transmission and infection rates, and other adverse clinical outcomes. We are unaware of any directives or health policies in Australian correctional settings regarding this practice, possibly because it has rarely been studied in prison sexual health research.

Nevertheless, we know that prisoners are at an increased risk of contracting bloodborne viruses in prison arising from blood-to-blood contact during injecting drug use, tattooing and violence [34]. Undertaking procedures such as inserting penile implants while in prison in the absence of proper infection control measures is fraught with potential risks such as infections at the site of insertion and acquiring bloodborne viruses. It is highly unlikely that prison authorities will provide prisoners with the necessary equipment to safely undertake penile implanting when more conventional practices such as tattooing equipment and clean injecting equipment is currently prohibited in most of the world's prisoners. In the absence of this, providing access to antiseptic solutions could provide some protection against infection. Our findings suggest that, at minimum, prison health education campaigns should include information on the risks associated with penile implanting in unsterile conditions. Prison health staff need to emphasise seeking treatment immediately if prisoners experience pain, swelling, redness and infection as a result of any artificial modifications to their penises.

A limitation of this study is that we had no way of validating the self-report, particularly in regard to the practice of interest to this study – penile implants. It is possible that some participants, particularly men, overestimated their sexual activities [35]–[37]. Conversely, some women may have under reported sexual activity. We were unable to determine how many prisoners agreed to participate only to receive the AUD$10 payment. We cannot exclude the possibility that some individuals provided socially desirable responses. However, this did not appear to be borne out by the response to sensitive questions such as engagement with sex workers, engagement in sex before the age of consent, and histories of sexually transmitted infections. Our study excluded prisoners who were being transferred between prisons, in court or hospital, or who were deemed ‘too dangerous’ to be interviewed by prison authorities, those who had insufficient English, and those who could not provide informed consent. As these findings are based on cross-sectional data, causal inferences cannot be made.

## Conclusion

Penile implants appear to be fairly common among prisoners and associated with prisoners engaging in other risky sexual and drug use practices. As most of these penile implants are inserted in prison, these men are at risk of acquiring blood borne viruses and infections. More research needs to be conducted to understand the extent and impact of the practice and harm reduction strategies developed to address this risk.

## References

[1] Brown D, Edwards J, Moore R (1988) The Penis Inserts of Southeast Asia: An Annotated Bibliography With an Overview and Comparative Perspectives. Berkeley, California: Center for South & Southeast.

[2] Fischer N, Hauser S, Brede O, Fisang C, Muller S (2010) Implantation of artificial penile nodules–a review of literature. The journal of sexual medicine 7 (11) 3565–71. 20102449 10.1111/j.1743-6109.2009.01659.x

[3] Lim KB, Seow CS, Tulip T, Daniel M, Vijayasingham SM (1986) Artificial penile nodules: case reports. Genitourinary medicine 62 (2) 123–5. 3721509 10.1136/sti.62.2.123 PMC1011913

[4] Loue S, Loarca LE, Ramirez ER, Ferman J (2002) Penile marbles and potential risk of HIV transmission. Journal of immigrant health 4 (2) 117–8. 16228768 10.1023/A:1014506811197

[5] Marzouk E (1990) Implantation of beads into the penile skin and its complications. Scandinavian journal of urology and nephrology 24 (3) 167–9. 2237292 10.3109/00365599009180852

[6] Kelly A, Kupul M, Aeno H, Neo J, Naketrumb R, et al (2012) More than just a cut: A qualitative study of penile practices and their relationship to masculinity, sexuality and contagion and their implications for HIV prevention in Papua New Guinea. BMC international health and human rights 12 (1) 10. 22818494 10.1186/1472-698X-12-10 PMC3520875

[7] Hull TH, Budiharsana M (2001) Male circumcision and penis enhancement in Southeast Asia: matters of pain and pleasure. Reproductive health matters 9 (18) 60–7. 11765401 10.1016/s0968-8080(01)90091-6

[8] Lee R, Norella B, Ragas R, Sibbaluca M, Tena C (2001) Between the thighs: penile circumcision, implants and sexual gadgets. Unpublished research. Manila: De La Salle University.

[9] Djajakusumah TS, Meheus A (2000) Artificial nodules of the penis: case report of an Indonesian man. Sexually transmitted diseases 27 (3) 152–3. 10726648 10.1097/00007435-200003000-00006

[10] Rothschild MA, Ehrlich E, Klevno WA, Schneider V (1997) Self-implanted subcutaneous penile balls–a new phenomenon in Western Europe. International journal of legal medicine 110 (2) 88–91. 9168326 10.1007/s004140050037

[11] Setiawan IM (2008) Social risk networks and HIV/AIDS among fishing boat crews in Benoa Port, Bali. Unpublished PhD Thesis. Chicago: Public Health Sciences, University of Illinois. 436 p.

[12] Thomson N, Sutcliffe CG, Sirirojn B, Sintupat K, Aramrattana A, et al (2008) Penile modification in young Thai men: risk environments, procedures and widespread implications for HIV and sexually transmitted infections. Sexually transmitted infections 84 (3) 195–7. 18192295 10.1136/sti.2007.028530

[13] Wardi N (2011) Young Male Prisoners Implanting Artificial Penile Nodules: “A Sign of Vulnerability or an Expression of Agency?”. Unpublished Masters of Arts in Development Studies thesis. The Hague, Netherlands: Institute of Social Studies. Available: http://thesis.eur.nl/theses/law_culture_society/iss/index/674680255/. Accessed 2012 May.

[14] Wilcher G (2006) Artificial penile nodules–a forensic pathosociology perspective: four case reports. Medicine, science, and the law 46 (4) 349–56. 10.1258/rsmmsl.46.4.349 17191640

[15] Hudak S, McGeady J, Shindel A, Breyer B (2012) Subcutaneous Penile Insertion of Domino Fragments by Incarcerated Males in Southwest United States Prisons: A Report of Three Cases. The journal of sexual medicine 9 (2) 632–34. 22081893 10.1111/j.1743-6109.2011.02551.x PMC3557851

[16] Beyrer C, Sripaipan T, Tovanabutra S, Jittiwutikarn J, Suriyanon V, et al (2005) High HIV, hepatitis C and sexual risks among drug-using men who have sex with men in northern Thailand. AIDS (London, England) 19 (14) 1535–40. 10.1097/01.aids.0000183122.01583.c7 16135908

[17] Serour F (1993) Artificial nodules of the penis. Report of six cases among Russian immigrants in Israel. Sexually transmitted diseases 20 (4) 192–3. 8211534

[18] Thorburn K, Allen T (1982) Penile Sperules [Letter to the Editor]. The American journal of forensic medicine and pathology 3 (3) 287. 10.1097/00000433-198209000-00021 7148785

[19] Rutty G (2001) Essentials of Autopsy Practice: Current Methods and Modern Trends. London: Springer.

[20] Tsunenari S, Idaka T, Kanda M, Koga Y (1981) Self-mutilation. Plastic spherules in penile skin in yakuza, Japan's racketeers. The American journal of forensic medicine and pathology 2 (3) 203–7. 7325129

[21] Tsunenari S, Yonemitsu K, Kanbe T, Kanda M (1984) How to identify the Yakuza, Japanese racketeers–their sociology, criminology and physical characteristics. Annals of the Academy of Medicine, Singapore 13 (1) 25–31. 6517501

[22] Nitidandhaprabhas P (1975) Artificial penile nodules: case reports from Thailand. British journal of urology 47 (4) 463. 1180995 10.1111/j.1464-410x.1975.tb04009.x

[23] Norton SA (1993) Fijian penis marbles: an example of artificial penile nodules. Cutis; cutaneous medicine for the practitioner 51 (4) 295–7. 8477613

[24] Wolf P, Kerl H (1991) Artificial penile nodules and secondary syphilis. Genitourinary medicine 67 (3) 247–9. 2071130 10.1136/sti.67.3.247 PMC1194682

[25] Primus Lake M (2004) Penile Modification and Its Impact to Sexual Health and Gender Relation: A Story from West Timor - Indonesia. *The XV International AIDS Conference: Abstract no. D11841*. Bangkok, Thailand.

[26] Butler T, Malacova E, Richters J, Yap L, Grant L, et al (2012) Sexual behaviour and sexual health of Australian prisoners. Sexual Health In Press. 10.1071/SH12104 23257020

[27] Butler T, Richters J, Yap L, Papanastasiou C, Richards A, et al.. (2010) Sexual health and behaviour of Queensland prisoners with Queensland and New South Wales comparisons. Perth and Sydney: National Drug Research Institute, Curtin University, Perth, and School of Public Health and Community Medicine, University of New South Wales, Sydney, Australia.

[28] Richters J, Butler T, Yap L, Kirkwood K, Grant L, et al.. (2008) Sexual health and behaviour of New South Wales prisoners. School of Public Health and Community Medicine, University of New South Wales, Sydney, Australia.

[29] Weeks MF (1992) Computer-assisted survey information collection: a review of CASIC methods and their implications for survey operations. Journal of Official Statistics 8 (4) 445–65.

[30] SPSS Inc. (2006) SPSS Version 15. Chicago, IL: SPSS Inc. [program]

[31] Smith AM, Rissel CE, Richters J, Grulich AE, De Visser RO (2003) Sex in Australia: the rationale and methods of the Australian Study of Health and Relationships. Australian & New Zealand Journal of Public Health 27 (2) 106–17. 14696700 10.1111/j.1467-842x.2003.tb00797.x

[32] Ware JE, Kosinski M, Keller SD (1998) SF-12: how to score the SF-12 physical and mental health summary scales, 3rd edition. Lincoln, RI: Quality Metric.

[33] Furukawa TA, Kessler RC, Slade T, Andrews G (2003) The performance of the K6 and K10 screening scales for psychological distress in the Australian national survey of mental health and well-being. Psychological Medicine 33: 357–62. 12622315 10.1017/s0033291702006700

[34] Butler T, Papanastasiou C (2008) National Prison Entrants' Bloodborne Virus and Risk Behaviour Survey Report 2004 & 2007. National Drug Research Institute (Curtin University) & National Centre in HIV Epidemiology and Clinical Research (University of New South Wales). Available: http://ndri.curtin.edu.au/local/docs/pdf/publications/R223.pdf. Accessed 2012 Jun.

[35] Alexander MG, Fisher TD (2003) Truth and consequences: Using the bogus pipeline to examine sex differences in self-reported sexuality. Journal of Sex Research 40: 27–35. 12806529 10.1080/00224490309552164

[36] Catania JA, Binson D, Canchola J, Pollack LM, Hauck W, et al (1996) Effects of interviewer gender, interviewer choice, and item wording on responses to questions concerning sexual behaviour. Public Opinion Quarterly 60: 345–75.

[37] Fisher TD (2007) Sex of experimenter and social norm effects on reports of sexual behavior in young men and women. Archives of Sexual Behavior 36: 89–100. 17187217 10.1007/s10508-006-9094-7

